# The Amphiregulin/EGFR axis has limited contribution in controlling autoimmune diabetes

**DOI:** 10.21203/rs.3.rs-3204139/v1

**Published:** 2023-08-03

**Authors:** Arielle Raugh, Yi Jing, Matthew L. Bettini, Maria Bettini

**Affiliations:** 1 Translational Biology and Molecular Medicine Program, Baylor College of Medicine, Houston, Texas, 77030, USA; 2 Department of Pediatric Endocrinology, Texas Children’s Hospital, Houston, Texas, 77030, USA; 3 Department of Pathology, University of Utah, Salt Lake City, Utah, 84112, USA

## Abstract

Conventional immunosuppressive functions of CD4+Foxp3+ regulatory T cells (Tregs) in type 1 diabetes (T1D) pathogenesis have been well described, but whether Tregs have additional non-immunological functions supporting tissue homeostasis in pancreatic islets is unknown. Within the last decade novel tissue repair functions have been ascribed to Tregs. One function is production of the epidermal growth factor receptor (EGFR) ligand, amphiregulin, which promotes tissue repair in response to inflammatory or mechanical tissue injury. Whether such pathways are engaged during autoimmune diabetes and promote tissue repair is undetermined. Previously, we observed upregulation of amphiregulin at the transcriptional level was associated with functional Treg populations in the non-obese diabetic (NOD) mouse model of T1D. We postulated that amphiregulin promoted islet tissue repair and slowed the progression of diabetes in NOD mice. Here, we report that islet-infiltrating Tregs have increased capacity to produce amphiregulin and both Tregs and beta cells express EGFR. Moreover, we show that amphiregulin can directly modulate mediators of endoplasmic reticulum (ER) stress in beta cells. Despite this, NOD amphiregulin deficient mice showed no acceleration of spontaneous autoimmune diabetes. Taken together, the data suggest that the ability for amphiregulin to affect the progression of autoimmune diabetes is limited.

## Introduction

Regulatory T cells (Tregs) are a unique subset of CD4 T lymphocytes that have a major role in preventing autoimmunity through regulation of antigen presentation, expression of immunosuppressive molecules, and creating competition for energy and resources^[1]^. In addition to these canonical functions, non-lymphoid tissue-resident Tregs can also secrete factors that promote tissue regeneration. These specialized tissue-Treg features are instructed by both TCR activation and tissue derived signals, such as IL-33, IL-18, and TGFβ^[2–4]^. Treg mediated tissue repair is driven in part through the secretion of growth factors, such as the epidermal growth factor receptor (EGFR) ligand, amphiregulin (Areg)^[2]^. In models of tissue injury, Tregs at the tissue site upregulate amphiregulin and induce tissue repair^[3–6]^. However, the role of amphiregulin during chronic tissue inflammation and damage has not been fully resolved, as the disease models explored to date largely focus on acute injury models. In one example, an induced model of acute skeletal muscle injury resulted in the accumulation of Tregs expressing amphiregulin within the injured muscle, with stellate stem cells responding to amphiregulin and repopulating myofibrils^[5]^. In addition, following influenza infection induced damage, Treg-derived amphiregulin acted to repair epithelial cells apart from their immunosuppressive functions^[3]^.

Type 1 diabetes (T1D) is a T lymphocyte mediated autoimmune disease that leads to the destruction of insulin producing beta cells within the pancreatic islets of Langerhans. Tregs are critical for protection against autoimmunity and can restrain autoimmune T cells to delay disease progression^[7]^. We had previously observed that functional Tregs upregulate amphiregulin at the transcriptional level as they enter the pancreatic islets in a non-obese diabetic (NOD) model of type 1 diabetes^[8,9]^. However, whether the amphiregulin/EGFR axis is engaged and can be protective during autoimmune diabetes was unclear. The potential role for this pathway in T1D is consistent with observed reduced levels of amphiregulin in recently diagnosed patients^[10]^, and decreased levels of another EGFR ligand, epiregulin, in individuals at a high risk of developing T1D^[11]^. Furthermore, the EGFR pathway has the potential to be broadly important in the context of T1D, as EGFR has been identified as a susceptibility gene for diabetic neuropathy and diabetes associated cardiac dysfunction^[12,13]^. We hypothesized, however, that amphiregulin could have a direct role in beta cell survival, based on the observations that beta cells require EGFR signaling for normal development and postnatal proliferation^[14]^, and that constitutive activation of EGFR on beta cells protects against streptozotocin induced diabetes^[15]^.

Here, we report that islet-infiltrating Tregs upregulate amphiregulin in the NOD mouse model of autoimmune diabetes. Moreover, we show that amphiregulin can have a direct effect on beta cells by reducing ER stress in *ex vivo* cultured islets. However, the deletion of amphiregulin in NOD mice does not change spontaneous autoimmune diabetes development, suggesting that any protective effects induced by amphiregulin are not sufficient to maintain or recover beta cell mass. Therefore, our data suggest that the amphiregulin/EGFR axis is not always effective in controlling inflammatory tissue damage. While potentially promising, the use of amphiregulin as a therapeutic target presents several challenges that will need to be addressed through further study.

## Results

### Islet-infiltrating Tregs upregulate amphiregulin

**Figure 1.**
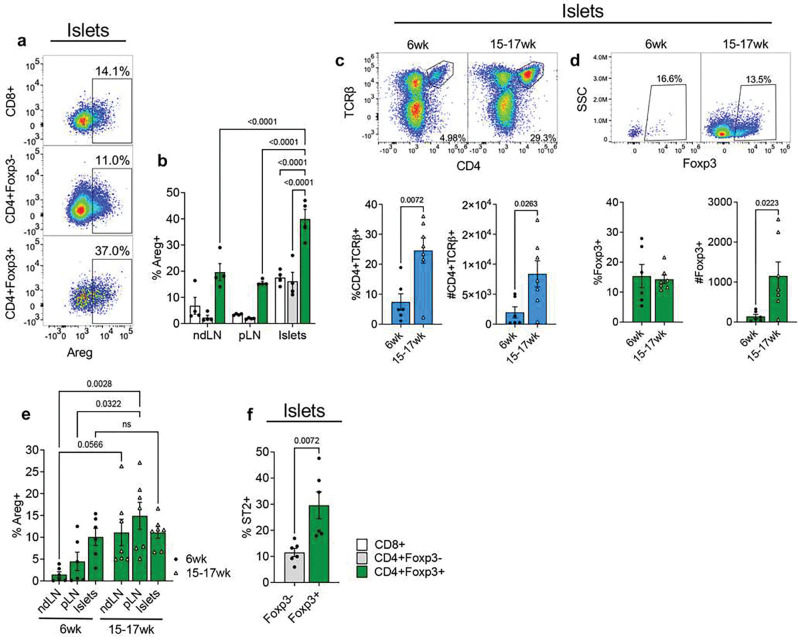
NOD islet Tregs are a major source of amphiregulin. **a)** Representative plots of Areg expression on *in vitro* stimulated CD8+ T cells, CD4 Teffs (CD4+Foxp3−) and Tregs (CD4+Foxp3) isolated from the islets of 14wk female NOD mice (n=4). **b)** Quantification of *in vitro* stimulated cells in non-draining lymph nodes (ndLN), pancreatic-draining lymph nodes (pLN), and islets. Two-way ANOVA with Tukey multiple comparison. **c)** Representative plots and quantification of CD4+TCRβ+ frequencies and cell count in younger (6wk, n=6) and older (15–17wk, n=7) NOD mice. Two-tailed unpaired t-test. **d)** Representative plots and quantification of Foxp3+ frequencies and cell count in younger (6wk, n=6) and older (15–17wk, n=7) NOD mice. Two-tailed unpaired t-test. **e)** *In vivo* Areg expression from ndLN, pLN, and islet Foxp3+ Tregs in younger (6wk, n=6) and older (15–17wk, n=7) NOD mice. Mice were injected with BFA, and amphiregulin production by Tregs was measured 6 hours later. Two-way ANOVA with Tukey multiple comparison. **f)** ST2 expression on CD4+Foxp3− and CD4+Foxp3+ cell populations isolated from islets of 6wk NOD mice (n=6). Two-tailed unpaired t-test. Data shown as Mean with SEM.

We had previously observed that islet-infiltrating CD4+Foxp3+ Tregs upregulate amphiregulin at the transcriptional level^[8,9]^. To confirm that amphiregulin was indeed expressed by islet-infiltrating Tregs at the protein level, we used flow cytometric analysis of T cells isolated directly from infiltrated islets or peripheral lymphoid organs and stimulated *in vitro*. Islet, pancreatic-draining lymph node, and non-draining lymph node T cells from pre-diabetic 14-week-old female NOD mice were analyzed for amphiregulin expression. First, our data show that Foxp3+ Tregs expressed amphiregulin at a greater frequency compared to Foxp3− CD4 Teffs and CD8 Teffs regardless of the tissue site (Figure 1a and 1b). Secondly, among Tregs, the highest frequency of amphiregulin expression was observed within the pancreatic islets (~40%+) (Figure 1b), suggesting that tissue resident Tregs have an enhanced capacity to express amphiregulin.

To determine whether Tregs actively produce amphiregulin *in vivo*, we treated NOD mice with brefeldin A to block amphiregulin secretion, and then analyzed intracellular amphiregulin directly *ex vivo*. In parallel, we sought to determine if amphiregulin expression by Tregs changed during disease progression by analyzing both young and old mice. While overall CD4 T cell frequency and number increased in older mice (15–17-week-old) compared to younger mice (6-week-old) (Figure 1c), Foxp3+ frequency remained consistent, although Foxp3+ cell number increased (Figure 1d), indicating an expected increase in the number of immune cells infiltrating into the pancreatic islets. Frequency of amphiregulin positive cells within the Foxp3+ islet population was unchanged overtime (~10%+), suggesting that islet resident Tregs maintain their capacity to produce amphiregulin throughout the course of disease (Figure 1e). However, older mice had a greater frequency of amphiregulin positive Tregs in both draining and non-draining lymph nodes compared to younger mice, suggesting systemic activation of regulatory T cells at later stages of disease.

Prior models of injury in which Treg derived amphiregulin was essential to tissue recovery showed co-expression of amphiregulin and the IL-33 receptor, ST2^[3]^, consistent with the ability for the IL-33/ST2 pathway to induce amphiregulin expression in Tregs^[16,17]^. To determine if islet Tregs similarly expressed ST2, we analyzed ST2 expression on islet CD4 T cells by flow cytometry. In congruence with previous reports^[18]^, a higher frequency of Tregs expressed ST2 compared to Teffs (Figure 1f). Taken together, these data show that islet resident Tregs express the canonical markers of tissue repair Tregs, amphiregulin and ST2.

### Beta cells express amphiregulin receptor EGFR

**Figure 2.**
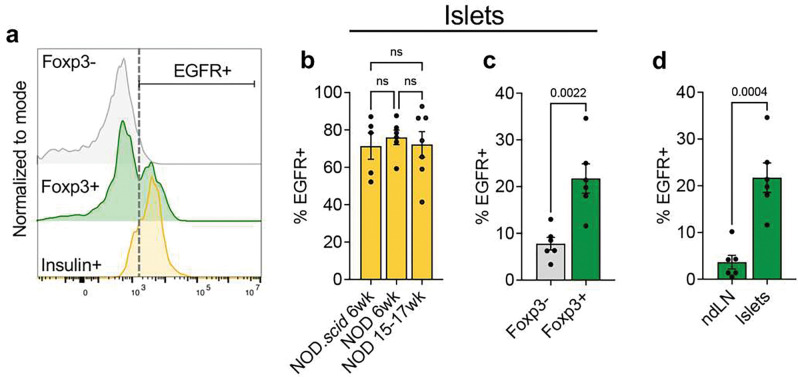
Beta cells and T cells within NOD islets express the amphiregulin receptor, EGFR. **a)** Representative histogram of EGFR expression on CD4+ Foxp3−/Foxp3+ cells and insulin+ beta cells from the islets of NOD mice. **b)** EGFR expression on beta cells (insulin+) from either NOD.*scid* (6wk, n=5), younger (6wk, n=6), or older NOD mice (15–17wk, n=7). One-way ANOVA with Tukey multiple comparison. **c)** EGFR expression on Teffs (CD4+Foxp3−) and Tregs (CD4+Foxp3+) within NOD young (6wk) islets (n=6). Two-tailed unpaired t-test. **d)** EGFR expression on Tregs (CD4+Foxp3+) from non-draining lymph nodes (ndLN) and islets from 6wk NOD mice (n=6). Two-tailed unpaired t-test. Data shown as Mean with SEM.

Once we defined Tregs as a major source of amphiregulin within the islets, we analyzed *ex vivo* expression of the amphiregulin receptor EGFR to determine the potential cellular targets of amphiregulin. We observed a relatively high level of EGFR expression on insulin positive beta cells (~73% EGFR+), and beta cell EGFR levels remained consistent between 6 week and 15–17-week-old NOD mice (Figure 2a and 2b). To determine whether inflammatory signals during T1D modify levels of EGFR expression on beta cells, we measured EGFR levels on beta cells from NOD.*scid* mice, which lack T and B cells and do not develop autoimmunity. As there was no difference in EGFR expression between NOD and NOD.*scid* mice (Figure 2b), we concluded that beta cell EGFR expression is stable regardless of inflammation, which suggested that beta cells could respond to amphiregulin throughout disease.

In addition to beta cells, a proportion of both CD4+ effector and CD4+Foxp3+ regulatory T cells within the islets expressed EGFR on their cell surface, although Tregs expressed significantly higher levels (~8% compared to ~22%) (Figure 2c). Therefore, it is possible that Tregs may utilize amphiregulin in a paracrine fashion during autoimmunity, as well as secrete amphiregulin to promote tissue repair^[19]^. Although levels of EGFR expressed on beta cells did not differ between normal and inflammatory conditions, previous studies suggested that EGFR expression on Tregs could be induced in response to inflammation^[19]^. To determine if EGFR expression is induced on Tregs in response to autoimmune stress, we compared EGFR levels on Tregs in the islets to the levels expressed by Tregs in the non-draining lymph nodes (Figure 2d). Tregs in the islets indeed showed higher levels of EGFR expression compared to the non-draining lymph nodes, indicating that Tregs upregulate EGFR in response to inflammation within the islets and thus have a better potential to respond to amphiregulin.

### Amphiregulin modulates BiP, a mediator of the UPR and ER stress in beta cells

The high demand for insulin production in beta cells results in transient endoplasmic reticulum (ER) stress in response to glucose stimulation^[20]^. However, under chronic ER stress conditions, such as those found during diabetes, the unfolded protein response (UPR) may lead to loss of beta cell function, and further unresolved ER stress can lead to beta cell death^[21,22]^. Importantly, amphiregulin was previously shown to be upregulated during beta cell recovery phase after transient hyperglycemia induced by deletion of the UPR sensor IRE1α^[23]^, suggesting a role for amphiregulin in modulating beta cell mass in response to ER stress. Thus, we asked if amphiregulin was directly capable of reducing ER stress in beta cells.

**Figure 3.**
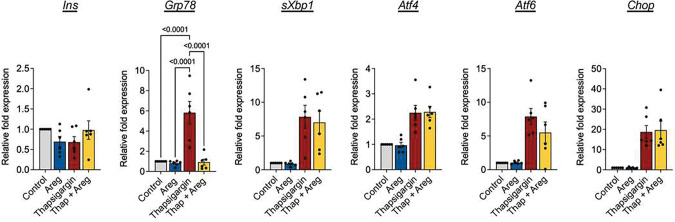
Amphiregulin reduces beta cell ER stress. qPCR analysis of cultured islets treated with thapsigargin. Islets from four NOD.*scid* mice were pooled and either cultured in 10% FBS RPMI with or without 100ng/mL recombinant mouse Areg and/or 5mM Thapsigargin for 7 hours. Expression relative to *Gapdh*. One-way ANOVA with Tukey multiple comparison. N=3, Data shown as Mean with SEM.

Using thapsigargin, a SERCA pump inhibitor, we induced ER stress in cultured NOD.*scid* islets, and measured transcription of genes commonly associated with the three arms of the UPR (Figure 3). Insulin transcript served as a control for similar beta cell distribution among the treatment groups. All of the assessed ER stress genes were upregulated in response to thapsigargin treatment, as expected. ER stressed islets were treated with recombinant amphiregulin protein to assess the potential for amphiregulin to modulate thapsigargin induced ER stress. Early downstream targets of the UPR (*sXBP1*, *ATF4*, and *ATF6*) were unchanged with the addition of amphiregulin, although *sXBP1* and *ATF6* did trend towards decreased transcription levels. In addition, transcription of the pro-apoptotic factor, *CHOP*, was unaltered by amphiregulin. However, transcription levels of the chaperone binding immunoglobin protein (BiP) (*Grp78*) were significantly downregulated with amphiregulin, showing that amphiregulin can directly act upon beta cells. While this evidence suggests that amphiregulin cannot completely prevent or reduce ER stress in *ex vivo* cultured islets, amphiregulin may still be able to moderate certain aspects of the UPR. As BiP is situated upstream of ER stress transcriptional changes, the return to basal *Grp78* levels in the amphiregulin treated group may indicate the return of beta cells to transient ER stress.

### Lack of systemic inflammation in amphiregulin deficient NOD mice

**Figure 4.**
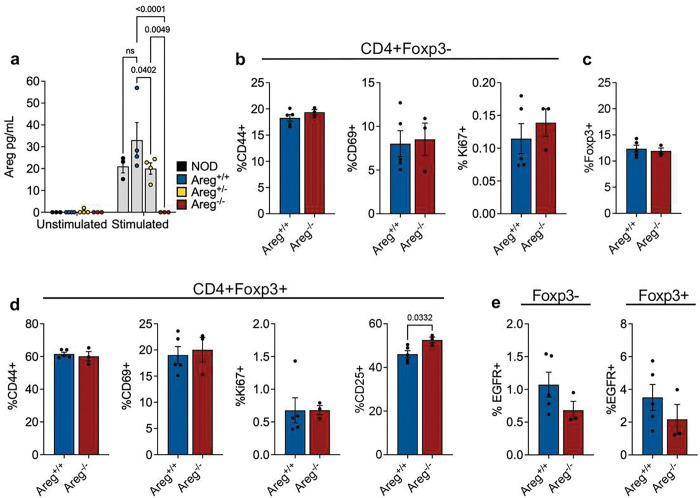
Amphiregulin deficiency in NOD mice does not lead to systemic immune dysregulation. **a)** CD4 T cells were enriched from pooled splenocytes and non-draining lymph nodes and cultured with or without PMA/Ionomycin (1ug/mL) for 24 hrs. Supernatant was analyzed using Areg ELISA. Two-way ANOVA with Holm-Sidak multiple comparison. NOD (n=3), NOD.Areg^+/+^ (n=4), NOD.Areg^+/−^ (n=4), NOD.Areg^−/−^ (n=3). **b)** Frequency of activation and proliferation markers on peripheral CD4+Foxp3− Teffs. Two-tailed unpaired t-test. **c)** Frequency of Foxp3+ Tregs in the periphery of NOD.Areg+/+ and NOD.Areg−/− mice. Two-tailed unpaired t-test. **d)** Frequency of activation and proliferation markers on peripheral Foxp3+ Tregs. Two-tailed unpaired t-test. **e)** Frequency of EGFR+ Teffs and Tregs in the periphery. Two-tailed unpaired t-test. **b-e)** 7–9wk female NOD Areg^+/+^ (n=5), Areg^−/−^ (n=3). Data shown as Mean with SEM.

To determine the physiological importance of amphiregulin in the development and progression of autoimmune diabetes, we backcrossed the amphiregulin mutation^[24]^ onto the NOD genetic background to create amphiregulin deficient NOD mice. Areg^Mcub(−/−)^ is a point mutation in the amphiregulin locus in a donor splice site of exon 1, causing the formation of a premature stop codon. Loss of amphiregulin production was confirmed by ELISA using enriched and *ex vivo* stimulated CD4 T cells (Figure 4a). To determine if this mutation within the amphiregulin gene led to global immune dysregulation on the susceptible NOD background, markers of T cell activation and Treg function were analyzed in the periphery of NOD.Areg^−/−^ female mice and wild type (NOD.Areg^+/+^) littermate controls. There were no differences in the level of activation of peripheral CD4 T effector cells based on the expression of CD44, CD69, or Ki67 (Figure 4b), and CD4+Foxp3+ Treg frequencies in amphiregulin deficient mice were similar to Tregs from control wild type mice (Figure 4c). Tregs from both groups also showed a similar level of activation based on CD44, CD69, and Ki67; however, there was a slight, but significant increase in CD25 expression in amphiregulin deficient mice (Figure 4d). Interestingly, there was a trend towards decreased EGFR levels on both Teffs and Tregs of NOD.Areg^−/−^ mice, possibly as a result of reduced positive feedback in response to amphiregulin binding (Figure 4e). Overall, these observations did not show systemic immune dysregulation or loss of Tregs in amphiregulin deficient animals.

### Amphiregulin expression does not impact development of autoimmunity

**Figure 5.**
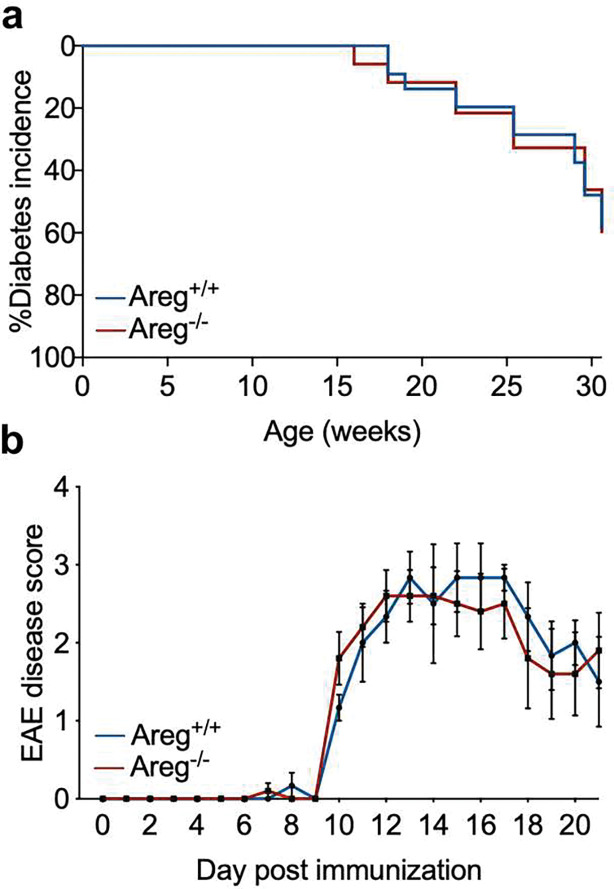
Amphiregulin deficiency does not impact development of autoimmunity **a)** Diabetes incidence of female NOD.Areg^+/+^ (n=23) and NOD.Areg^−/−^ (n=17) mice. Log-rank Mantel-Cox. **b)** 7wk female C57BL/6.Areg^+/+^ (n=3) and C57BL/6.Areg^−/−^ mice (n=5) were immunized with MOG_35-55_/CFA and monitored daily for EAE symptoms. Two-way ANOVA with Bonferroni multiple comparison.

To fully determine whether loss of amphiregulin in NOD mice leads to reduced beta cell survival and accelerated diabetes, we monitored female NOD.Areg^−/−^ mice and wild-type littermate controls for the development of spontaneous autoimmune diabetes (Figure 5a). Surprisingly, we detected no difference in diabetes incidence or progression between the two groups. The similarity in diabetes development between NOD.Areg^+/+^ and NOD.Areg^−/−^ mice indicates that amphiregulin does not have a role in autoimmune diabetes disease progression.

Finally, to determine if the limited role of amphiregulin during NOD spontaneous diabetes development was organ specific, and if amphiregulin could have a functional role in other types of autoimmunity, we induced experimental autoimmune encephalomyelitis (EAE) in 7-week-old female C57BL/6.Areg^−/−^ and littermate wild-type control mice. EAE was induced with a MOG_35-55_ peptide emulsion and pertussis toxin as described previously^[25]^. Similar to our results obtained from NOD diabetes model, amphiregulin seemed to have no impact on the development of EAE (Figure 5b). In congruence with others who have suggested that amphiregulin is dispensable for protective Treg function during EAE^[26]^, we also saw that the severity of EAE disease remained unchanged between Areg^+/+^ and Areg^−/−^ mice.

## Discussion

Regulatory T cells that infiltrate or reside within tissues are often uniquely adapted to be effective within their environment. In some situations, Tregs with repair functions exhibit a unique repair transcriptional signature, indicating a specialized subpopulation or lineage^[27,28]^. Based on single-cell transcriptomics, tissue-resident Tregs differentiate from a lymphoid tissue phenotype into subpopulations that have conserved transcriptional programs^[29]^. Some of these programs involve unique functions such as maintenance of tissue homeostasis as well as damage repair, and include the EGFR ligand amphiregulin. Here we show that pancreatic Tregs can express amphiregulin in addition to other known immunosuppressive molecules^[1]^. Furthermore, islet-resident Tregs express ST2, the receptor for the alarmin cytokine IL-33. The expression of ST2 and amphiregulin on islet-infiltrating Tregs implies that these cells can respond to tissue damage signals as part of a repair program, in accordance with previous findings^[3,16,17]^. Importantly, we show that beta cells express high levels of the amphiregulin receptor, EGFR, suggesting that beta cells can respond to amphiregulin. In fact, the presence of recombinant amphiregulin during thapsigargin induced ER stress in cultured islets reduced *Grp78* (BiP) transcription, a regulator of the unfolded protein response pathway. All of these observations point to amphiregulin as a potentially important mechanism employed by islet-infiltrating Tregs to curb beta cell destruction. Surprisingly, however, amphiregulin deficiency in NOD mice did not significantly alter autoimmune diabetes incidence or progression.

The lack of impact of amphiregulin deficiency on autoimmune diabetes led us to question whether amphiregulin also fails to protect in other autoimmune models. Using C57BL/6 amphiregulin deficient mice, we saw that loss of amphiregulin did not impact the severity of EAE induced by MOG_35-55_ peptide immunization. This observation is consistent with a recent study showing that amphiregulin knock-down in Treg adoptive therapy does not impede their ability to suppress EAE^[26]^. The protective role of amphiregulin seems to be disease or context dependent, as amphiregulin is effective in controlling immune responses during autoimmune colitis^[19]^ and pristane-induced lupus nephritis^[30]^.

The effectiveness of amphiregulin in tissue repair may be dependent on the target cell. In mice, adult beta cells show a relatively low proliferative capacity^[31]^. This low regenerative potential may limit the ability for amphiregulin to repair damaged beta cells at a rate that can compete with the damage caused by autoimmune assault during T1D. Furthermore, in certain disease models the target of amphiregulin during injury are stem-like cells that undergo induced differentiation into tissue cells to help support tissue repair. For example, amphiregulin expressing Tregs that accumulate in muscle fibers following acute injury prompt the differentiation of muscle satellite cells^[5]^. Such stem-like pre-cursor cells for beta cells have not been identified. In contrast to acute muscle injury, chronic infection with the parasite *Toxoplasma gondii*, increases amphiregulin expression in muscle Tregs, but does not prevent tissue damage^[32]^. In the case of *T. gondii*, Tregs appear to increase the ratio of IM/M1 (inflammatory monocytes/pro-inflammatory macrophages) to M2 macrophages (pro-regenerative macrophages) preventing skeletal muscle fiber regeneration. Therefore, amphiregulin has limited function under certain inflammatory conditions.

As the field looks to innovative ways of preventing, attenuating, or reversing T1D disease progression, several methods have become promising. One of these approaches is to attenuate the autoimmune attack on beta cells by reducing the number of activated autoreactive T effectors. Teplizumab – the first FDA approved immunotherapy to delay T1D – is a monoclonal antibody against CD3 that showed increased frequencies of unresponsive CD8 T cells in treated individuals^[33]^. In addition, current clinical trials are using expanded autologous Tregs re-infused into patients to suppress autoimmune responses^[34]^. However, a second and complementary strategy in T1D therapies is directed towards improving beta cell survival and restoring beta cell mass and function, either through the protection of beta cells against autoimmune attack, or beta cell regeneration. Whether Treg therapy could be harnessed to enhance beta cell survival in addition to inducing autoimmune suppression is unknown, but is an active area of investigation. Apart from endogenous Treg functions, cells could be enhanced to achieve additional functional competency. For example, Tregs could be re-engineered in order to increase antigen specificity either through exogenous CAR or TCR^[35]^. Treg expression of amphiregulin can be driven by alarmin cytokines (i.e. IL-33 and IL-18) that are produced by injured tissues^[3,4,36]^. Consistent with the role of IL-33, tissue resident Tregs often have high expression of the IL-33 receptor, ST2^[37]^. A recent study has overexpressed ST2 on human Tregs to successfully induce TCR-independent reparative mechanisms, in particular increasing the production of amphiregulin^[17]^. Furthermore, treatment of T1D patient PBMCs *in vitro* with IL-33 increased Treg frequencies and FOXP3 expression^[38]^, consistent with previous reports indicating an ability of IL-33 to enhance Treg induction^[39]^. Thus, with these new technologies emerging, it is of great interest to understand whether the IL-33/amphiregulin/EGFR axis can act directly or indirectly to retain beta cell function during T1D, and whether amphiregulin should be further pursued as a therapeutic target for T1D.

During autoimmune diseases such as T1D, many tissue resident Tregs are antigen specific^[40]^ and express markers associated with enhanced immunosuppressive functions^[41]^; however, it is unclear whether pancreatic Tregs also have a non-immunological purpose within the islets. Our findings provide evidence for a population of islet-infiltrating Tregs that express amphiregulin and ST2; markers associated with tissue repair Tregs. However, while amphiregulin can mitigate beta cell ER stress, analysis of NOD amphiregulin deficient mice suggest that amphiregulin does not overtly impact autoimmune diabetes incidence. Why the amphiregulin/EGFR pathway is not effective in supporting beta cell function is unclear, but could point to another reason for the inability of Tregs to effectively control anti-beta cell responses. As our study did not investigate the outcomes of other tissues often damaged by hyperglycemia (kidneys, blood vessels, heart), amphiregulin could have an effect in improving other comorbidities commonly associated with T1D^[13]^.

Lastly, there are several limitations of our study that should be considered before we rule out amphiregulin as an effective pro-beta cell factor. The first being that the effects of amphiregulin during autoimmunity were studied using mice that have a global deletion of amphiregulin. The amphiregulin mutation may have resulted in activation of compensatory mechanisms, for example through upregulation of other EGFR ligands. Alternatively, Tregs are known to compensate for lost regulatory functions via upregulation of alternative suppressive mechanisms^[42]^. Secondly, although islet Tregs produce amphiregulin and beta cells express EGFR, we did not investigate whether EGFR is actively signaling in beta cells. It is possible that post-translational processing of amphiregulin, or downstream EGFR signaling is changed during T1D leading to sub-optimal amphiregulin/EGFR axis activation. Additionally, this study focused on the effect of amphiregulin in pancreatic islets during autoimmune diabetes and did not explore additional tissues commonly impacted by diabetic hyperglycemia. Thus, while amphiregulin does not impact overt diabetes development, the impact amphiregulin may have on other aspects of T1D cannot be ruled out. Additional studies will be necessary to determine whether there are deficiencies in beta cell response to EGFR ligands during autoimmune diabetes and inflammatory tissue damage.

## Materials and Methods

### Mice

All mice were housed and used according to IACUC approval at Baylor College of Medicine Houston, TX and The University of Utah Salt Lake City, UT. B6.Cg-*Areg*^*Mcub*^ *Rhbdf2*^*cub*^/J were obtained from The Jackson Laboratory (Strain #003628) and crossed with C57BL/6 mice to remove the *Rhbdf2*^*cub*^/J mutation. B6.Areg^Mcub^ (i.e. Areg^−/−^) mice were bred heterozygously and littermates were used for experiments. The B6.Areg^Mcub^ strain was backcrossed to NOD mice for 10 generations to produce the NOD.Areg^−/−^ mice. NOD.Areg^−/−^ mice were bred heterozygously and littermates were used for experiments. All experiments were performed in accordance with relevant guidelines and regulations and reported following recommendations in the ARRIVE guidelines.

### Islet Isolation

Single islet isolation was performed as previously described^[43]^. Briefly, pancreata were perfused via the bile duct with Collagenase 4 (Worthington Biochemical), incubated at 37° for 30 min, and islets were picked with a p10 pipette under a dissecting scope. Islets were dissociated into single cell suspension with enzyme free dissociation buffer (Gibco) for 15 minutes at 37° with vortexing every 5 minutes. Dissociated islets were washed with HBSS before proceeding to analysis.

### Flow Cytometry

#### Cell phenotype analysis

Isolated cells were stained with antibodies for surface markers at room temperature for 15 minutes. Intracellular staining protocol was modified from eBioscience Foxp3 staining kit. Cells were fixed in 2% methanol free paraformaldehyde for 30 minutes at room temperature. Intracellular staining was done in Foxp3 permeabilization buffer (eBioscience) overnight at 4°. Cells were analyzed by flow cytometry on a BD Biosciences LSRFortessa. Data was analyzed using FlowJo 10 analysis software (BD Biosciences).

#### In vivo cytokine quantification

*In vivo* cytokine production was evaluated as described previously^[44]^. Briefly, Brefeldin A (BFA) powder was dissolved in DMSO (20mg/mL) and stored at −20°^C^. Immediately before use, concentrated BFA stock was diluted to a working concentration (0.5mg/mL) in PBS and 500 uL was injected via tail vein. Six hours post injection, mice were euthanized, and islets and lymph nodes were analyzed by flow cytometry on Cytek Aurora (Cytek Biosciences).

#### In vitro T cell activation

Islets from 14-week-old female NOD mice were isolated and stimulated at 37° in RPMI supplemented with 10% FBS for 5 hours with PMA/Ionomycin (1ug/mL), Brefeldin A (5ug/mL), and Monensin (2uM). Intracellular amphiregulin was detected using biotinylated amphiregulin antibody (R&D Systems BAF989) followed by conjugated streptavidin for 30 min on ice.

#### Antibodies

anti-amphiregulin (Polyclonal, R&D Systems, Biotinylated), anti-CD4 (GK1.5, Biolegend, BD Biosciences, PerCP-Cy5.5, BV605, BUV496), anti-CD5 (53-7.3, Biolegend, BV510, PerCP-Cy5.5), anti-CD8 (53-6.7, Biolegend, PE), anti-CD25 (PC61, Biolegend, BV785), anti-CD44 (IM7, Biolegend, AF700), anti-CD69 (H1.2F3, Biolegend, PerCP-Cy5.5), anti-CTLA-4 (UC-10-4B9, Biolegend, PECy7), anti-EGFR (ICR10, Abcam, FITC), anti-Foxp3 (FJK-16s, eBioscience, eF450), anti-ICOS (C398.4A, Biolegend, AF647), anti-insulin (T56-706, BD Pharminogen, AF647), anti-ST2 (DIH4, Biolegend, PE), Streptavidin (Biolegend, APC, BV711), anti-TCRβ (H57-597, Biolegend, APC-FIRE).

### *Ex vivo* islet ER stress

Pancreatic islets were isolated from NOD.*scid* mice as described above. Whole islets were then cultured in RPMI supplemented with 10% Fetal Bovine Serum (FBS) for 7 hrs. Additional stimulation conditions included recombinant mouse Areg (100ng/mL) (Recombinant mouse Amphiregulin, Carrier-free, Biolegend), Thapsigargin (5uM) (Enzo Life Sciences), or mAreg plus Thapsigargin. After 7 hrs, total RNA was extracted following the manufacturer’s protocol from Qiagen RNeasy kit. First strand cDNA synthesis was performed using the manufacturer’s protocol from High-Capacity Reverse Transcriptase kit (Applied Biosciences). The following targets were analyzed by qPCR (*Insulin, Grp78, sXBP1, CHOP, ATF4, ATF6, GAPDH*) using a QuantStudio6 (Applied Biosystems). Primers for each target are listed in Table 1.

### Amphiregulin ELISA

Splenocytes from NOD.Areg^+/+^, NOD.Areg^+/−^, and NOD.Areg^−/−^ mice were enriched for CD4+ cells using the AutoMACS Pro Separator (Miltenyi Biotec) *possel* program and stimulated for 24 hrs in standard RPMI media supplemented with 10% FBS and with the addition of PMA and Ionomycin (1ug/mL). Supernatant was collected and used in the mouse amphiregulin duoset ELISA (R&D Systems) following the manufacturer’s protocol. Absorbance was measured on a microplate reader (BioTek Synergy H1) at 450nm and 540nm. Readings at 540nm were subtracted from readings at 450nm for wavelength correction, and concentrations were calculated using GraphPad Prism 9.0 Sigmoidal Curve based on recombinant amphiregulin standard curve.

### Diabetes Monitoring

Female NOD.Areg^+/+^ and NOD.Areg^−/−^ mice were monitored weekly for overt diabetes using urinalysis (Diastix, Ascensia Diabetes Care). If urinalysis was positive, blood glucose readings using a glucometer and glucometer strips (Freestyle Lite) was performed. Mice were determined to be diabetic with one reading >400mg/dL or two consecutive readings of >300mg/dL.

### Experimental Autoimmune Encephalomyelitis (EAE)

EAE was induced as previously described^[25]^. Briefly, 4mg/mL Complete Freund’s Adjuvant was used to make a peptide emulsion of MOG35-55 at a 1:1 ratio the day prior to injecting mice. On day zero, 7-week-old female C57BL/6.Areg^+/+^ and Areg^−/−^ mice were immunized with 100ul MOG_35-55_ peptide/CFA emulsion s.q. into each flank (total 200ul), and injected with 200ul of 1 ug/ml *Bordetella pertussis* toxin diluted in PBS i.p. On day two, each mouse was injected with an additional 200ul of 1 ug/ml *Bordetella pertussis* toxin. Beginning on day seven post first injection, mice were scored for motor symptoms daily using the following scale.

Score 0: No obvious physical motor differences are observed.

Score 1: Complete flaccidity of the tail or hind limb weakness (not both).

Score 2: Both limp tail and hind limb weakness or partial paralysis.

Score 3: Total hind limb paralysis. The mouse can no longer use hind limbs to maintain rump posture or walk.

Score 4: Hind limb paralysis and front limb weakness/paralysis. With the total loss of movement in hind limbs, the mouse drags itself only on its forelimbs. Mice appear alert and feeding, but do not move around the cage.

Score 5: Moribund. Mice are not feeding, not alert, and close to death.

### Statistics

Data were entered and graphed using GraphPad Prism 9.0. Data are presented as Mean with standard error of mean (SEM) unless otherwise noted. Statistical tests used are indicated in Figure Legends.

## Figures and Tables

**Table 1.** Primer sequences of targets used in qPCR.

Target		Sequence
Insulin	Forward	GTCAAGCAGCACCTTTGTGGTTCC
	Reverse	ACAATGCCACGCTTCTGCTG
Grp78	Forward	TGCTGCTAGGCCTGCTCCGA
	Reverse	CGACCACCGTGCCCACATCC
sXbp1	Forward	GAGTCCGCAGCAGGTGC
	Reverse	CAAAAGGATATCAGACTCAGAATCTGAA
Atf4	Forward	GCCGGTTTAAGTTGTGTGCT
	Reverse	CTGGATTCGAGGAATGTGCT
Atf6	Forward	GATGCAGCACATGAGGCTTA
	Reverse	CAGGAACGTGCTGAGTTGAA
Chop	Forward	CGGAACCTGAGGAGAGAGTG
	Reverse	CGTTTCCTGGGGATGAGATA
Gapdh	Forward	TGCACCACCAACTGCTTAG
	Reverse	GGATGCAGGGATGATGTTC

## Data Availability

The datasets generated during and/or analyzed during the current study are available from the corresponding author on reasonable request.
